#  Transition from in-hospital ventilation to home ventilation: process description and quality indicators

**DOI:** 10.3205/000259

**Published:** 2017-12-19

**Authors:** Marc Kastrup, Benjamin Tittmann, Tanja Sawatzki, Martin Gersch, Charlotte Vogt, Max Rosenthal, Simone Rosseau, Claudia Spies

**Affiliations:** 1 Department of Anesthesiology and Operative Intensive Care Medicine, CCM/CVK, Charité – Universitätsmedizin Berlin, corporate member of Freie Universität Berlin, Humboldt-Universität zu Berlin, and Berlin Institute of Health, Berlin, Germany; 2 Department for Hematology, Oncology and Palliative Care – Sarcoma Centre Berlin-Brandenburg, Bad Saarow, Germany; 3 Freie Universität Berlin, School of Business & Economics, Department of Information Systems, Chair of Business Administration, Berlin, Germany; 4 Klinik Ernst von Bergmann Bad Belzig gGmbH, Bad Belzig, Germany

**Keywords:** quality indicator, in-hospital, out-hospital, intensive care medicine, long-term ventilation, weaning, process management, multiprofessional

## Abstract

The current demographic development of our society results in an increasing number of elderly patients with chronic diseases being treated in the intensive care unit. A possible long-term consequence of such a treatment is that patients remain dependent on certain invasive organ support systems, such as long-term ventilator dependency. The main goal of this project is to define the transition process between in-hospital and out of hospital (ambulatory) ventilator support. A further goal is to identify evidence-based quality indicators to help define and describe this process.

This project describes an ideal sequence of processes (process chain), based on the current evidence from the literature. Besides the process chain, key data and quality indicators were described in detail. Due to the limited project timeline, these indicators were not extensively tested in the clinical environment.

The results of this project may serve as a solid basis for proof of feasibility and proof of concept investigations, optimize the transition process of ventilator-dependent patients from a clinical to an ambulatory setting, as well as reduce the rate of emergency re-admissions.

## Zusammenfassung

Durch die demographische Entwicklung bedingt werden zunehmend ältere Patienten mit chronischen Begleiterkrankungen intensivmedizinisch versorgt. Als eine mögliche Folge einer solchen Intensivbehandlung kann bei diesen Patienten eine chronische Abhängigkeit von apparativen Organersatzverfahren resultieren, welche im Bereich der Beatmung in einer Langzeitbeatmung resultiert. Ziel dieses durch das BMBF geförderte Projekt „Beatmungspflege@home“ ist die Definition des Überleitprozesses von der klinischen Beatmung in die außerklinische Beatmung und die Entwicklung von evidenzbasierten Qualitätsindikatoren um diesen Prozess der Überleitung transparent darzustellen.

Im Projektverlauf wurde eine Soll-Prozesskette, unter Berücksichtigung der aktuellen Literatur in Bezug auf die Evidenzlage, erstellt. Anhand dieser Soll-Prozesskette wurden parallel die Kennzahlen und Qualitätsindikatoren detailliert definiert und konsentiert. Aus Gründen der begrenzten Projektlaufzeit konnte kein ausführlicher Feldtest zur Evaluation der Praxistauglichkeit durchgeführt werden.

Die Projektbeteiligten hoffen, dass die Ergebnisse des Projektes eine solide Grundlage darstellen, um im Anschlussprojekt einen „proof of concept“ untersuchen zu können, um damit den Überleitprozess in die außerklinische Beatmung qualitativ zu verbessern und somit unnötige Klinikeinweisungen zu verhindern.

## Introduction

Current demographic developments have led to an increase in the number of elderly patients with chronic diseases in intensive care units (ICUs) that require a complex management. A possible consequence of ICU treatment is the long-term dependency on caregivers and/or organ support systems, such as mechanical ventilation. Aside from central nervous system disorders, such as paraplegia or disruption of respiratory centers, chronic obstructive pulmonary disease (COPD) is one of the most common ailments resulting in long-term ventilator dependency.

There is evidence that the care of long-term ventilation patients is entirely inadequate [1], [2]. The prevalence of COPD is projected to rise to 11% by the year 2050 [3]. Apart from the severe impairments on quality of life, the economic burden is estimated to be as high as 2.6 to 8.2 billion Euros per year [4].

The number of high-risk patients treated in the ICU, such as elderly and multi-morbid patients, is expected to rise significantly. The inadequate treatment of these patients can easily lead to cognitive and physical impairments, negatively impact long-term outcomes, and ultimately result in care dependency [5].

Following ICU treatment, the rate of weaning failure from ventilators is higher for elderly and/or multi-morbid patients. After managing the acute illness, these patients often require a transfer to specialized weaning units for long-term ventilator support, and subsequent initiation of outpatient respiratory management.

Throughout the past years, both the rate of successful weaning and the rate of long-term ventilator support have remained relatively constant. Data published by Schönhofer et al. [6] in 1999 and by Mifsud Bonnici et al. [7] in 2016, both show that between 60 and 65% of patients treated in a specialized unit could be successfully weaned, while 25% still required intermittent ventilation following discharge.

Additionally, with a one-year survival rate of 50–60% [6], [7], the long-term prognosis of these patients is severely impaired.

A decreasing average length of stay on the ICU highlights the transition from the ICU setting to an outpatient management, resulting in an increased demand for out-of-hospital ventilation support.

There are several possible reasons for this development: (1) the introduction of the DRG system, (2) the attempt to decrease nosocomial infections through shorter inpatient treatment times, (3) the goal to increase patient comfort by transitioning to outpatient management, (4) technological advances in outpatient respiratory medicine, (5) improved survival rate of acute severe illness leading to higher rates of chronic course of disease, (6) the increasing capabilities of out-of-hospital institutions to provide respiratory and intensive care [8].

Many private practices are not equipped with the necessary expertise to competently manage patients in need of ventilation therapy, as this has traditionally taken place in the clinical setting. In recent years, numerous ambulatory care services were founded to meet the rising demand for these highly specialized outpatient interventions.

There is an increasing trend for this type of care to take place in residential settings, even in living communities.

Patients and their relatives often welcome such concepts of long-term care. Given certain professional and structural requirements, most clients feel safe and report an increased sense of participation and autonomy [9].

The field of ambulatory respiratory care has yet to establish basic, common qualitative standards. The influence of external control mechanisms in the ambulatory setting of highly specialized care is currently very limited [10].

The cooperative project “Beatmungspflege@Zuhause” (Bea@Home) was funded by the German Federal Ministry of Education and Research (BMBF) between 2013 and 2016. The primary goal is to define the transition process of ventilated patients from inpatient clinical treatment to outpatient care, according to the standards of evidence-based medicine, along with the development of appropriate quality indicators for such a transition.

A collaboration of eight project partners, from the fields of science and industry, collaborated to evaluate various options of technology-assisted patient care, and the implementation of data networks into the living and nursing environments of patients dependent on long-term respiratory support.

Apart from using digital instruments to document medical and nursing interventions, a major goal of this joint project was the development of telemedical applications, such as video-based rounds (eConferences) and the upload of treatment data to control centers.

Similar to the management of other conditions, it is in the interest of legislative and funding bodies to require a standardized, cross-sectoral clinical pathway for the field of respiratory care. Following a complete and detailed definition of the process chain and the quality indicators, such a concept must be extensively tested for feasibility in routine practice. Unfortunately, Bea@Home was limited in this regard due to the short duration of the project.

## Methods

Several partners in the joint project are already integrated in the healthcare process, or are able to provide technical assistance in the future.

The project partners are as follows: Linde Remeo Deutschland GmbH, The School of Business & Economics of the Freie Universität Berlin, CC 07 Charité – Universitätsmedizin Berlin, CC 12 Charité – Universitätsmedizin Berlin, German Institute of Applied Nursing Research e.V. (dip), Linde AG, T-Systems International GmbH, CIBEK technology + trading GmbH, and Prosystem AG int. Healthcare Consulting. All partners of this joint project have actively participated in generating the results presented here. During the initiation phase, various work packages were defined and distributed among the partners, according to their relevant area of expertise.

An early review of the literature revealed a number of interaction opportunities amongst the interdisciplinary project partners. To facilitate an effective flow of communication, several measures were taken. First, multiple observerships (network meetings) were organized to introduce members of the project to the local structure of, as well as the processes within, each participating institution. Second, several project meetings were organized, which were attended by some or all of the partners. These meetings focused on individual topics among the different work packages, and served to update all involved parties on the overall project development. According to the industrial, economic, or medical background of the participants, differing perspectives quickly emerged regarding viable options for the transition process. There was critical need for developing an interdisciplinary blueprint for the proposed process, in terms of care, administration, and communication. To enable optimal interdisciplinary communication, as well as process transparence for all involved parties, the cross-sectoral care process was modelled via an event-driven process chain (EPC).

Several iterative steps were integral to EPC development. The current state of care was used in the EPC as a basis for the development. Then, using a stepwise approach, a consented version of the target state was established. The individual work packages were modified in parallel, in accordance to the current/target process chain. During development, project partners with a clinical or nursing background recommended the planning of an ethics workshop to adequately account for the needs and wishes of the patients. This workshop took place in March 2014, and was attended by all project partners, who decided to adopt the MEESTAR model for the duration of the project. The MEESTAR model (societal, organizational, and individual perspectives) serves to address ethical issues regarding age-appropriate assistance systems [11]. From an ethical perspective, the following issues were identified for further consideration during the course of the project: (1) extensive patient education, (2) development and implementation of an IT-system to secure all relevant treatment data, (3) broaden consent forms to encompass additional information, such as financial aspects, (4) provide an extensive, structured transfer conference, without a time limit, and (5) regular ethics-based case conferences in regular intervals, even after patient transfer.

After the definition of a target process chain, and based on the results from the literature review, a first draft of the key figures and quality indicators was developed. These were refined in subsequent project meetings, until a consensus was reached.

Due to the limited timeframe of this project, only one patient was included in the field testing. For this reason, no information can be provided on the actual feasibility of the project. Further investigations involving a large number of patients undergoing the transition process can enable further refinement of the key figures and quality indicators, which are necessary before the start of a “proof of concept” evaluation.

## Results

### Target process chain

The figure in Attachment 1 illustrates the complete target process chain for the transition of care of patients requiring ventilation support into an out-of-hospital setting. The target process chain is grouped into three segments. The first (upper) segment describes in-hospital patient management, including the transfer to an ICU specialized in weaning patients from mechanical ventilation. The middle segment describes processes surrounding outpatient ventilation support in facilities specialized for ambulatory weaning. The lower segment describes the patient care in their home setting. The necessary documents and information, as well as the utilized information systems, are visualized in the middle and lower segments.

The accompanying process interfaces (documents, information systems, and standardized single services) are presented according to their implementation time, from left to right, along the time axis. Structures/components that were identified as relevant during all parts of the process are shown on the right. 

### Quality indicators/key figures

Our literature review revealed only few publications dealing with the positive effects of quality indicators on the transition of patients with ventilation support into home care. The Association of the Scientific Medical Societies in Germany (AWMF) published guidelines and recommendations for weaning from ventilation [12], as well as for the non-invasive and invasive ventilation of patients with chronic respiratory failure [13]. The German Respiratory Society (DGP) has released a position paper on the management of mechanically ventilated patients in the home care environment in 2008 [9]. These publications, along with expert opinion expressed by project participants, served as the basis for defining 10 main quality indicators for the process of transitioning ventilated patients from the clinic to their home environment. Each of these main indicators was divided into several sub-sections. The quality indicators are as follows:

1. Weaning process in the intensive care unit (physician care)
2. Defining indications in the weaning unit (physician care)
3. Outpatient physician management (physician care)
4. Patient‘s wishes and goals of therapy (ethics)
5. Patient information and informed consent (ethics)
6. Transfer conference (discharge management)
7. Individual assistive needs (discharge management)
8. Multimodal therapeutic concept (therapeutic concept)
9. Individualized nursing care planning (nursing)
10. Patient safety, quality of life, and patient-reported outcomes (PRO)

An in-depth definition for each quality indicator can be found in Attachment 2.

Specific key figures and ranges were defined for each of the above areas, including ideal place and time of documentation.

As a next step of the joint project, a field test on 10–15 patients was planned to evaluate and refine each indicator as necessary. At the end of the project, and after receiving approval by the ethics and data protection committees, one patient went through the defined pathways from the intensive care unit to the weaning unit and into outpatient respiratory care. The strict inclusion and exclusion criteria (only adult patients fulfilling the indications for continuous ventilation via tracheostomy) were major reasons for the low patient number, as well as the fact that no other patient matching the inclusion criteria was transferred to an outpatient facility that was participating in the joint project during the recruitment period. For this patient, all the indicators could be recorded as planned, and the audio-visual follow-up rounds (eConferences) were successfully implemented via a telemedical approach. The electronic patient record utilized for the outpatient mechanical ventilation was an important factor enabling the telemedical communication. The team on the weaning unit could digitally control and manage patient data and the various therapeutic recommendations. This served as a basis for efficient communication between treatment teams, ensuring well-organized expert consultations by enabling real-time interaction with the course of management.

The establishment of hospital networks via telemedical technologies might promote patient supervision, reduce the number of visits to a general practitioners, and possibly influence the number of hospital admissions. Casavant and colleagues [14] demonstrated a positive effect of telemedical consultations (videoconferences) on hospital admissions and emergency room visits. Aside from audio-visual communication, the current project had additional access to the electronic medical records used in the outpatient respiratory center. This provided physicians with real-time access to relevant information on certain indicators of quality, including ventilator setting and current medication. According to the users, this form of interactive teleconsultation with patient chart access provided significant advantages over a “classical” audio-visual consultation.

Ethical aspects were considered in the development process of the key figures and the quality indicators at various stages of the process chain. For example, the overall treatment goals under consideration of the patient’s preferences, in addition to medical indications, should be defined by patients and relatives prior to discharge to the ambulatory setting. To get a long-term robust data structure, the quality indicators assessed quality of life at pre-defined time intervals: at discharge from the hospital, one month after discharge, and then every 6 months.

## Discussion

To establish transparency and to ensure baseline quality, the goals of this project were to define the process of transition of patients requiring long-term ventilator support to an outpatient setting, as well as to define relevant key figures and quality indicators. In 2009, the DGP published the S2 guideline for non-invasive and invasive mechanical ventilation for the treatment of chronic respiratory failure [13], which was revised in 2017. Its publication addressed an increasing rate of out-of-hospital mechanical ventilation, aiming to provide guidance to the increasingly diverse groups caring for these patients. The guideline includes “recommendations for implementation” for the transition process. To facilitate the real-world application of the recommendations, the coordination group for home mechanical ventilation also published the “Recommendations for Invasive Home Mechanical Ventilation” in 2011 [15]. The recommendations focused on patients with weaning-failure following acute treatment on an intensive care unit.

The underlying causes for long-term mechanical ventilation have not been adequately considered in previous work related to the transition of ventilated patients into the outpatient setting. The current guidelines for the management of delirium, analgesia and sedation on the intensive care unit [16] recommend the daily definition of sedation goals, as well as the assessment of sedation depth using a valid assessment tool at least once per shift. This recommendation aims to prevent protracted intensive care unit stays and weaning failures. Apart from sedation management, the treatment of respiratory failure and appropriate strategies for weaning are additional factors affecting long-term prognosis. Girard et al. [17] conducted a study on 336 intubated ICU patients, and showed that mortality could be significantly reduced by implementing both daily spontaneous awakening and daily spontaneous breathing trials, with the objective of possible extubation. Several studies could demonstrate the negative long-term effects of early deep sedation on outcomes, even several years after intensive care [18], [5], and in fact, some authors recommend not using any sedatives at all [19]. These aspects were carefully considered during the development of quality indicators in this project.

Research by Dybwik et al. [20] in 2010 revealed large discrepancies in the use and availability of home mechanical ventilation among different Norwegian counties. Missing data did not allow for an in-depth cause analysis, but the authors proposed that physician experience and the availability of specialized services are possible reasons for these differences. It is likely that the situation in Germany is equally diverse [21]. The quality indicators defined in this joint project set the basis for the development of a database that enables the objective measurement and comparison of regional differences in regards to the care of patients requiring long-term mechanical ventilation. Patient privacy is a crucial consideration when using such a database. If the quality indicators are to be implemented on a large scale, there must be regulations regarding data access for the various involved parties at different treatment time points. A complex data access model with different access levels will manage data availability, which can be used for process optimization while still providing patient data access to caregivers directly involved in the transitioning process. A joint database, accessible to all involved parties, serves as the foundation for such a data access model. The feasibility of such an IT-based case management system to navigate between routine clinical access and privacy barriers has been demonstrated in the successful application of the care portal “Ambulanzpartner” (outpatient partner). This project, published by Funke and colleagues, was designed for patients with amyotrophic lateral sclerosis (ALS), and analyzed the prescription of therapeutic accessories and aids in four ALS centers, comparing them according to their respective different category and funding agency [22]. They found large differences in the prescription rate of assistive devices for communication, orthoses, and motorized wheelchairs [22]. A long-term goal of such case management systems should include standardization of care, and the provision of safe and high-quality products for the patients.

A number of novel technological concepts have been introduced to the market to provide support to elderly patients and those in need of care, such as smart home applications, smart living, or smart health [23]. Although the functionality of individual appliances could be shown, it remains unclear whether, and to what extent, these various systems are able to interact and communicate in terms of an integrated application system. A wide-spread implementation depends on a structured combination of services and technological solutions [23], [24], with a special emphasis on privacy and data protection aspects. The joint project “Bea@Home” does not establish clinical treatment standards regarding, for example, the prescription of therapeutic appliances, but rather proposes quality standards for the process of transition of patients between different parts of the healthcare system. This will serve as the central basis for the further development of IT-based care structures for the various proposed applications (technological solutions and services).

Furthermore, several ethical issues are encountered during the treatment and management of patients requiring long-term mechanical ventilation. Implications of advance directives, especially regarding quality of life, have been recently discussed in various media outlets. Such issues require a case by case decision-making, taking into account the patient wishes, age, and underlying morbidity that has led to the dependency on mechanical ventilation. Data show a large cross-country variance within Europe in regards to the utilization of outpatient mechanical ventilation. Patients with a pulmonary disease in need of mechanical ventilation live, on average, less than a year after initiation of (home-) ventilation. Patients with a restrictive ventilation issues, especially the thoracic wall, live between 6 to 10 years after beginning home ventilation, while patients with a neuromuscular disorder with the need for outpatient mechanical ventilation generally survive over 6 years post-initiation [25], [26]. Patients with an underlying COPD have a significantly increased mortality, especially when the resting oxygen demand is elevated [27]. Although length of survival is an important outcome measure, changes in quality of life specific to the various underlying diseases must also be taken into consideration. A 2016 meta-analysis by MacIntyre et al. [28] reviewed studies containing health-related quality of life (HRQL) measures for patients receiving home mechanical ventilation. Patients suffering from COPD, which make up around 30% of European patients receiving outpatient ventilation, were explicitly excluded from the analysis due to the lack of comprehensive international regulations, and the fact that Canada does not recommend home ventilation for this patient collective. The authors concluded that most of the included studies considered the HRQL of patients on home ventilation is “good”. Home ventilation had a positive effect on mental testing results when the underlying disorder was of a predominantly physical-functional nature. The rate of hospital admissions and the total length of hospital stay were also decreased. Although there was no uniform assessment method, many of the studies found caregivers had high rates of stress, especially family members [28]. Future studies must determine to what extent various technological aids influence or reduce these burdens for the caregivers.

Windisch et al. [29] demonstrated an improvement in HRQL scores after initiation of home mechanical ventilation, regardless of the underlying disease. The improvement in quality of life seems to be age-dependent, as patients over the age of 75 that were treated with non-invasive mechanical ventilation due to chronic hypoventilation showed no improvement in quality of life after 6 months [30]. These issues must be discussed with patients and their relatives, as soon as a possibly invasive, long-term home mechanical ventilation is indicated. Recognizing the central importance of proper indication and quality of life, the project “Bea@Home” integrated these topics in the previously mentioned ethics workshop. Since the mortality rates of patients receiving invasive home mechanical ventilation can vary depending on the underlying disease, defining patient preferences and discussing the possibility of limiting, or deescalating, treatment strategies is strongly recommended, both verbally and in writing.

A 2007 survey of European respiratory care units by Nava et al. [31] identified a large variability, and limitations, in the practice of obtaining advance directives. Formal end-of-life decisions were available for a total of 21.5% of patients (n=1,292 of 6,008). Among the patients with known end-of-life directives, the therapy was to be maintained at current levels in 23% of cases, 34% had “do not resuscitate/do not intubate” orders, and 31% of patients wished non-invasive mechanical ventilation to be set as the maximum possible form of ventilation therapy. Of these, all 473 patients that were responsive took part in the decision-making, and the caregiver team was involved in decisions regarding all the patients that were unable to articulate their wishes. This study underlines broad national and international heterogeneity of end-of-life decision making, and highlights the need for clearly formulating and documenting these advance directives in such cases.

## Outlook

The results of the joint project “Bea@Home” provide a framework for large interdisciplinary and multi-centric field studies. All steps necessary for transitioning patients from inpatient to outpatient settings have been defined, as well as quality indicators and key figures for these individual steps. If the quality indicator prove to be reliable in clinical routine, they will provide several options for improving the management of patients receiving out-of-hospital mechanical ventilation. After establishing a common basis for data collection, financial data can be directly linked to properly documented interventions (e.g. pay for transparency) [32].

Data collection could further serve as a prerequisite for quality certifications of facilities providing outpatient mechanical ventilation. This could be expanded to include in-hospital acute care, weaning units, mechanical ventilation facilities, as well as outpatient mechanical ventilation, so that this data serves as an important element in governance architecture [33]. The results of this project may also provide a basis for the drafting of contracts between individual health insurance agencies and healthcare providers. The quality indicators can be implemented and assessed on different levels and at different stages of development.

As part of a uniform value scale (German: “einheitlicher Bewertungsmaßstab”, EBM) system for general practitioners and specialist physicians, the quality indicators could be implemented to establish quality premiums in the outpatient setting.

In a first step, payment could be partially coupled to data collection that enables an evaluation of the quality of care (*pay for transparency*). The next step would include the introduction of quality-based payment (*pay for performance*). The most sophisticated and complex implementation strategy would see a result-oriented payment system (*pay for outcome*) [32].

The trend towards digitalization and increased connectivity has not only fundamentally changed hospital practices, but also profoundly influenced various features in the outpatient sector, such as patient monitoring, telemedical rounds, robot-assisted nursing systems, and assistive technology for daily living. The joint project “Bea@Home” has introduced an initial approach to such interconnected concepts via the implementation of audio-visual conferences. The proof of concept stage will shed light on its large-scale practicability.

Many aspects in the outpatient treatment of mechanically ventilated patients remain problematic. While the nursing care is performed by specialized nursing services, the active management of the long-term ventilation strategies remain the responsibility of general practitioners, professionals who rarely possess specialized training in mechanical ventilation. The frequent involvement of emergency services personnel often results in an unnecessarily high rate of hospital readmissions, creating a revolving door effect. A regular indication reassessment for mechanical ventilation, a careful adjustment of ventilation parameters according to patient requirements, and a focus on patient wishes and advance directives, can all pose major challenges under such circumstances. By establishing audio-visual rounds and electronic documentation systems, the participants of the project hope to improve the standard of care in this increasingly common transition process.

## Notes

### Competing interests

The authors declare that they have no competing interests.

### Funding

This project was funded by the German Federal Ministry of Education and Research (BMBF, registration number: 16SV6061). 

### Authorship

Marc Kastrup and Benjamin Tittmann share the first authorship.

Simone Rosseau and Claudia Spies share the last authorship. 

### Acknowledgement

The authors want to thank the following persons for their valuable help in the preparation of this article:

- Dr. med. Peter Kalin
  Medical Director Region Europe Central Linde Gas Therapeutics GmbH
  Linde Healthcare
  Oberschleißheim, Germany
- Antje Kassin
  Nursing Director REMEO Deutschland
  Linde Remeo Deutschland GmbH
  Mahlow, Germany
- Marian Muschert
  Global Nurse Coordinator REMEO GGC Healthcare Global Marketing & Commercial Excellence
  Linde AG Linde Healthcare
  Pullach, Germany

## Abbreviations

AAPV = General Outpatient Palliative Care

AVK = audio-visual communication

CABS = Charité Center for Outpatient Mechanical Ventilation

CIRS = critical incident reporting system

COPD = chronic obstructive pulmonary disease

DIVI = German Interdisciplinary Association of Critical Care and Emergency Medicine

ePA = electronic patient file

IT = information technology

MDK = Health Insurance Medical Service (Medizinischer Dienst der Krankenkassen)

NIV = non-invasive ventilation

PA = patient file

QI = quality indicator

SAPV = specialized ambulatory palliative care

SBT = spontaneous breathing trial

SF 36 = short form health survey

SOP = standard operating procedure

SRI = self reporting initiative (health survey)

WZ = weaning center

## Supplementary Material

Target process chain for the transition of care of patients requiring ventilation support into an out-of-hospital setting

Quality indicators
