# Supplementary material for: Transition from in-hospital ventilation to home ventilation: process description and quality indicators
Source: Ger Med Sci. 2017 Dec 19;15:Doc18. doi: 10.3205/000259 (PMC5738500; doi:10.3205/000259)

Treatment Process Chain CC-LRD-Home

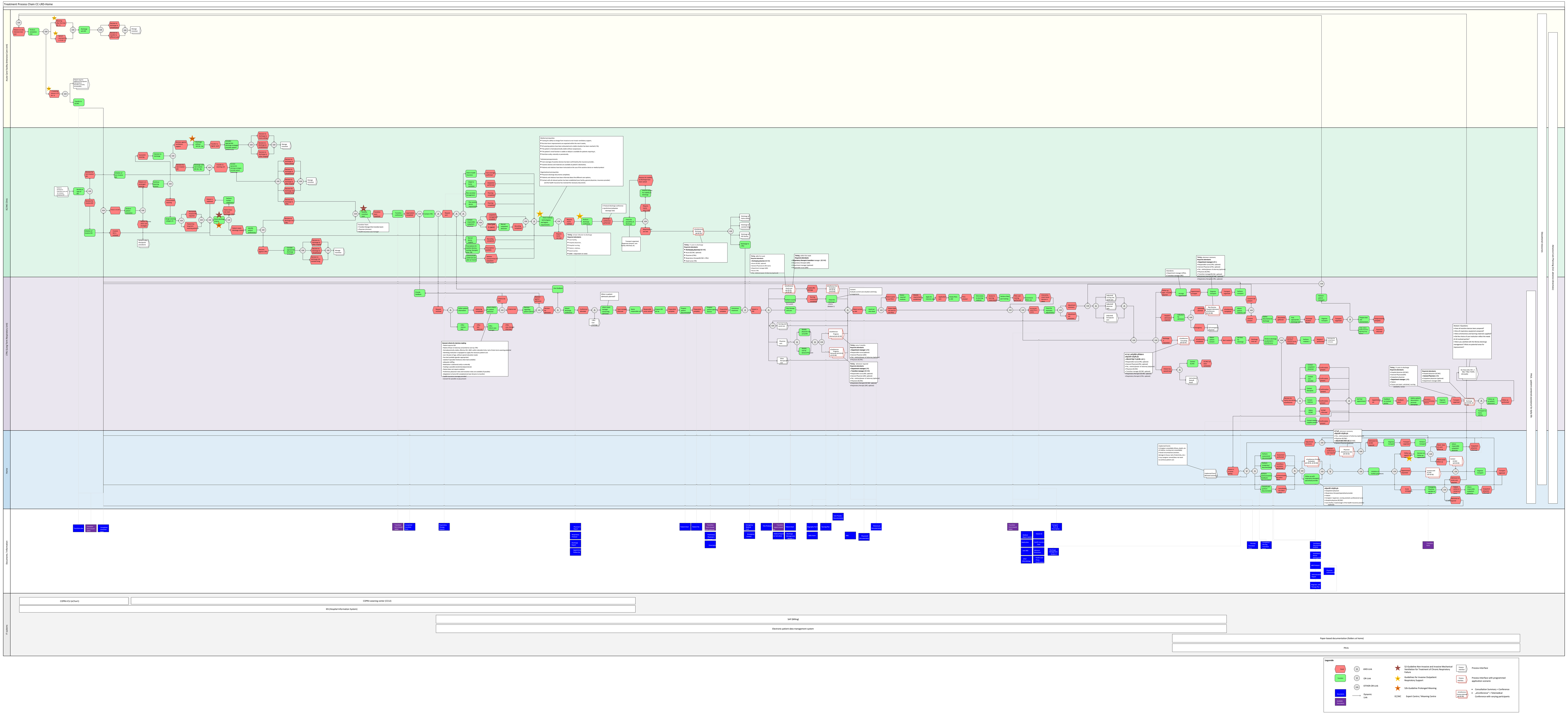

Supplement: Target process chain for the transition of care of patients requiring ventilation support into an out-of-hospital setting [file GMS-15-18-s-001.pdf]
