# Supplementary material for: Transition from in-hospital ventilation to home ventilation: process description and quality indicators
Source: Ger Med Sci. 2017 Dec 19;15:Doc18. doi: 10.3205/000259 (PMC5738500; doi:10.3205/000259)
Supplement: Quality indicators [file GMS-15-18-s-002.pdf]

1. Quality Indicator I

Weaning Process in the Intensive Care Unit

Dimension: Physician care

1.1 Monitoring of sedation and delirium via appropriate scores.

1.2 Weaning prerequisite checklist assessed daily and documented in patient record.

1.3 Performance and documentation of protocol-based spontaneous breathing trials (SBT).

1.4 Patients with prolonged weaning (category 3) and impending weaning failure are treated on a unit with certified qualifications for the management of prolonged weaning, or are being transferred to such a unit. The structural transfer to outpatient ventilation is managed by the treating clinic, given the necessary expertise, or in cooperation with a facility specializing in outpatient mechanical ventilation.

Reasoning and Definition:

- In patients with primary acute or acute on chronic respiratory failure, verification is required for a structured weaning process before transfer into the outpatient setting.
- Based on DIVI-Indicator IV: weaning protocol/concept in combination with sedation goals. Every patient receiving controlled ventilation should be assessed daily for weaning prerequisites and/or successful weaning. This has to be viewed in context with DIVI-Indicator QI II (monitoring of sedation, analgesia and delirium) which provides guidance on the scoring and documentation of sedation. SBTs must be documented.
- The weaning category must be documented in the (electronic) patient file and in the discharge notes.
- Patients are transferred into the outpatient setting, sometimes without fulfilling the necessary criteria.
- Patients' ability to wean has to be assessed in the context of freedom from delirium and unnecessarily deep sedation (monitoring!).
- The medical requirements must be fulfilled.
- The physician establishing the indication for mechanical ventilation must have expertise in the management of long-term ventilated patients.

  ↓

  Reports for every patient must be provided in the setting of examinations by the MDK ("German Health Insurance Medical Service") → was the treatment adequate? Adherence to QIs? (if not, recourse claims might result).

  ↓

  Reports are accessible to the parties involved in the consent discussions (Bea@Home QI 4).
- Frequent indication reassessment for ventilation throughout the weaning process in the weaning facility (WF) to avoid unnecessary mechanical ventilation.

Attachment to: Kastrup M, Tittmann B, Sawatzki T, Gersch M, Vogt C, Rosenthal M, Rosseau S, Spies C. Transition from in-hospital ventilation to home ventilation: process description and quality indicators. GMS Ger Med Sci. 2017;15:Doc18. DOI: 10.3205/000259

Key Figures:

Key Figure	Degrees of Freedom	Comments
• Monitoring of sedation and delirium via appropriate scores? (QI II)	• Yes/No<br>• Score	No transfer if results are positive
• Weaning trials according to SOPs were undertaken	• SBT undertaken and documented<br>• Protocol for weaning was followed	SOP of each facility/ SBT-algorithm
• Criteria for “Prolonged Weaning“-category fulfilled? (incl. DIVI-Indicator QI IV adherence)	• 3 SBTs undertaken and documented or >7 days since first SBT	SOP of each facility
• Weaning category documented in patient record/discharge letter?	• Yes/No	
• Treatment unit has expertise in the transfer to outpatient mechanical ventilation	• Yes/No	Written documentation!

2. Quality Indicator II

Defining Indications in the Weaning Unit

Dimension: Physician Care

2.1 Criteria for weaning category “prolonged weaning” group 3 C (weaning failure) are present and documented in patient record.
2.2 Possibility of transition to NIV has been evaluated and the results documented in patient record.
2.3 Indication for mechanical ventilation has been assessed by a specialist consultant with written justification and signature.
2.4 Possibility of mechanical ventilation and current treatment in the outpatient setting ascertained by physician and documented in patient record.
2.5 Patient is free of delirium at discharge (DIVI-Indicator QI II).
2.6 No administration of parenteral sedatives within 7 days prior to discharge (time of last administration must be documented).
2.7 Discharge notes must mention the responsible weaning unit or facility for outpatient mechanical ventilation by name.
2.8 Recommendations for follow-up have been documented in patient record.
2.9 Next follow-up appointment has been documented in patient record.
2.10 Regular follow-ups have taken place and have been documented in patient record.
2.11 Documentation on follow-up examination is present and accessible.

Reasoning and Definition:

- Patients receiving outpatient mechanical ventilation are a vulnerable population with complex treatment needs.
- To ensure a continuous, high quality patient treatment, the responsible general practitioner, the weaning facility, and possibly the homecare physician need to be known prior to discharge; name and contact information must be mentioned in the discharge notes, which must be accessible to everyone involved in the treatment (ePA); this ensures continuity of treatment without loss of information.
- Status of delirium, sedation and analgesia monitoring (DIVI-Indicator QI II), as well as possible medication adjustments and the current ventilation status (incl. e.g. the temporal progression of spontaneous breathing episodes), are assessed during physician contacts.

Key Figures:

Key Figure	Degrees of Freedom	Comments
• Presence of weaning category (3C) with appropriate documentation	• Yes/No	
• Evaluation for transition to NIV with appropriate documentation	• Yes/No	
• Specialist indication for home mechanical ventilation	• Yes/No	Documentation with justification by named physician
• Viability of ventilation support, and current treatment in outpatient setting	• Yes/No	Documentation in patient record (PA) by named physician
• Free of delirium at discharge	• Yes/No	Documentation in PA
• No parenteral sedatives given within 7 days prior to discharge	• Yes/No	Documentation in PA
• Responsible weaning unit / outpatient mechanical ventilation facility named in discharge note	• Yes/No	Documentation in discharge note
• Recommendations for follow-up documented	• Yes/No	Documentation in PA
• Appointment for follow-up made	• Yes/No	Documentation in PA
• Regular follow-ups have occurred	• Yes/No	Documentation in PA
• Physician notes for follow-up examinations are present	• Yes/No	Documentation in PA

3. Quality Indicator III

Outpatient Physician Management

Dimension: Physician Care

3.1 Name and contacts of responsible General Practitioner mentioned in patient record and discharge note.

3.2 Physician handover occurred.

3.3 Handover process documented in (e)PA.

3.4 Outpatient physician contacts

Reasoning and Definition:

- Analogous to the DIVI-Indicator QI VIII (structured documentation of talks with the next of kin), the communication between treating physicians and the patient’s next of kin is considered to be of great importance. Contact information of next of kin must be available to all treatment facilities.

Key Figures:

Key Figure	Degrees of Freedom	Comments
• Name and contact information of patient’s General Practitioner documented in patient record and discharge note	• Yes/No	Discharge note with required information is present and accessible
• Physician handover during patient transfer	• Yes/No	Documentation of discharge, outpatient follow-up or telephone call
• Contents of handover documented	• Yes/No	Documentation in (e)PA
• Outpatient physician contacts documented in the PA	• Yes/No	Documentation in (e)PA

4. Quality Indicator IV

Patient’s Wishes and Goals of Therapy

Dimension: Ethics

4.1 Patient wishes have been documented in the patient record.

4.2 Goals of therapy have been documented in notes and the patient record.

4.3 Documentation of structured patient/caregiver talks

4.4 Reassessment of patient wishes and goals of therapy in regular intervals, i.e. at least annually.

Reasoning and Definition:

- Patients have to be informed of the impact that a discharge into the outpatient setting will have on their lives – chances, risks, limitations; for people who have had no prior contact with patients in similar situations, it is hard to fathom the implications of being care-dependent – possibly having strangers in one‘s home, living with the constant risk of life-threatening events, e.g. ventilator failure, blockage by secretions, etc.
- These issues need to be clarified in one or more meetings; patients must be aware of the different treatment possibilities and alternatives in order to ensure informed decision making by the patient and/or next of kin.
- The meeting(s) must take place in certified weaning facilities before discharge into invasive outpatient mechanical ventilation. Time of meeting, participants and talking points, and patient concerns must be documented. Informed consent must be provided in written form, be signed by the responsible physician, and be accessible to all persons involved in the patients’ care (analogous to DIVI QI VIII).
- The goals of acute treatment must be known to the patient and treatment team (palliation? Return to baseline status prior to discharge?) depending on the underlying disease and its severity.
- As these goals represent a dynamic process, they must be reassessed <u>and documented</u> regularly.
- Possible discussion points:
  - Current status
  - Current treatment goal and patient wishes
  - Updates to advance directives
  - Formulation of goals/prognosis by the provider
- Updated advance directives can be helpful.

Key Figures:

Key Figure	Degrees of Freedom	Comments
• Patient wishes documented in the patient file	• Yes/No	Documentation in (e)PA
• Goals of therapy documented in notes and/or patient record	• Yes/No	Documentation in physician's note or (e)PA
• Documentation of structured talks	• Yes/No	Meeting with patient and/or next of kin or power of attorney, if patient is unable to make decisions
• Reassessment of patient wishes and therapy goals at least once a year	• Yes/No	Documentation in (e)PA, first reassessment within 1 year

5. Quality Indicator V

Patient Information and Informed Consent

Dimension: Ethics

5.1. Time point and participants documented in patient record.
5.2. Contents of patient information and possibly patient concerns documented in patient record.
5.3. Patient provides written informed consent, signed by the treating physician.

Reasoning and Definition:

- Treatment wishes are documented in written or audio-visual form, and accessible to all persons involved in the patient's care at all times (ePA: e.g. in the form of an advance directive, physician's notes, audio-video message, etc.).

Key Figures:

Key Figure	Degrees of Freedom	Comments
• Time point and participants documented in PA	• Yes/No	Documentation in (e)PA
• Contents and patient concerns documented	• Yes/No	Documentation in physician's note or (e)PA
• Written informed consent, signed by responsible physician	• Yes/No	Documentation in (e)PA

6. Quality Indicator VI

Transfer Conference

Dimension: Discharge Management

6.1. Time point, participants and contents of the transfer conference have been documented.
6.2. Protocol signed by the transfer manager and accessible.
6.3. Cross-sectoral patient record available for all participants.

Reasoning and Definition:

- Discharge management represents a challenging process, demanding the consideration of various specific requirements.
- Before the first discharge into outpatient mechanical ventilation, a transfer conference needs to be held, involving all disciplines and institutions involved (patient or power of attorney, next of kin, transfer manager + treating physician + nursing + social worker of discharging facility for prolonged weaning and outpatient mechanical ventilation; outpatient nursing, ventilation provider; outpatient physician; payer and/or MDK); a cross-sectoral, functional electronic patient record (ePA) is created and implemented.

Key Figures:

Key Figure	Degrees of Freedom	Comments
• Time point and participants of transfer conference in PA	• Yes/No	Documentation in (e)PA
• Protocol signed by transfer manager	• Yes/No	Documentation in physician's note or (e)PA
• Cross-sectoral ePA created and accessible to all parties	• Yes/No	Documentation in (e)PA

7. Quality Indicator VII

Individual Assistive Needs

Dimension: Discharge Management

7.1. Individual assistive needs evaluated according to checklist.
7.2. Checklist is assessed and adjusted for current needs.
7.3. Technical prerequisites for discharge are met and have been documented.

Reasoning and Definition:

- Patients receiving outpatient mechanical ventilation require a number of assistive measures, which must be individually tailored and be available in a timely manner.
- Checklists ensure seamless requirement planning, and will have to be regularly reassessed and modified.
- The assistive measures should enhance patient autonomy.
- The individual assistive needs are identified for improved patient autonomy.

Key Figures:

Key Figure	Degrees of Freedom	Comments
• Individual assistive needs assessed via checklist	• Yes/No	Checklist available in (e)PA
• Checklist assessed and adjusted for current needs	• Yes/No	Documentation in (e)PA
• Technical prerequisites for discharge are met and have been documented	• Yes/No	Documentation in (e)PA

8. Quality Indicator VIII

Multimodal Therapeutic Concept

Dimension: Therapeutic Concept

8.1 Individualized multimodal therapeutic concept has been created and documented.

8.2 Provision of treatment documented in patient record.

8.3 Evaluation of provision and treatment effectiveness has been documented .

8.4 Adjustment of concept when therapeutic goals are not met.

Reasoning and Definition:

- Continuously ventilated patients require intensive therapy, and possibly – depending on type and stage of their disease – a connection to specialized outpatient palliative care (SAPV) or general outpatient palliative care (AAPV).
- Therapeutic goals must be clearly defined and, if not met, the therapy or goals must be adjusted.

Key Figures:

- Treatment and regular assessments of therapeutic goals are documented in the ePA .
- A multimodal therapeutic concept has been created (esp. physical therapy, speech therapy, occupational therapy and possibly AAPV/SAPV).

Key Figure	Degrees of Freedom	Comments
• Individual multimodal therapeutic concept created and documented	• Yes/No	Therapeutic concept in ePA
• Treatments documented in patient record	• Yes/No	Documentation in PA
• Assessment of provision and effectivity of treatments documented	• Yes/No	Documentation in ePA

9. Quality Indicator IX

Individualized Planning of Nursing Care

Dimension: Nursing

9.1. Qualified nursing personnel (according to guideline recommendation).
9.2. Individualized planning of nursing care has taken place and has been documented.
9.3. Assessment of provision and effectivity has been documented.
9.4. Adjustment of planning if nursing goals are not met.
9.5. Weaning facility is available for contact and contacts are being documented.

Reasoning and Definition:

- In the outpatient setting, nursing management is of utmost importance, as physician-based treatment cannot be delivered with the same frequency as in the clinical setting.
- The role of the nursing staff in patient observation and treatment thus carries increased weight. Especially when it comes to assessment of ventilation status (e.g. potential for weaning) and the patient's status of analgesia, delirium and sedation (QI II).
- Patient observation by nursing staff has good potential to improve detection of weaning candidates. There is a need for defined parameters to advance the weaning process in suitable patients. Regular outpatient follow-ups and appointments at the weaning facility can monitor this process; additional contact is possible when needed (e.g., when the patient's status is worsening!).
- A highly qualified nursing staff is an absolute requirement to assess potential, as well as problems.
- Nursing efforts must be tailored to the individual needs and resources, taking into account the patient's wishes and therapeutic goals.

Key Figures:

- A sound, individualized nursing plan is being implemented and documented in the ePA.
- A multimodal, individualized concept for management of secretions is being implemented and documented in the ePA.
- The nursing staff assesses and documents analgesia, sedation and delirium status, and consults the physician when scores fall out of the target range or adjusts medication within pre-defined limits.
- The nursing staff is sufficiently qualified to take over management of a patient receiving mechanical ventilation (see the "Recommendations for Invasive Home Mechanical Ventilation").

Key Figure	Degrees of Freedom	Comments
• Nursing staff sufficiently qualified	• Yes/No	% of nursing staff with specialized skills
• Individualized nursing plan documented	• Yes/No	Documentation in PA
• Evaluation of provision and effectivity documented	• Yes/No	Documentation in PA, report on nursing status
• Adjustment of planning when nursing goals are not met	• Yes/No	When goals were not met, was the nursing plan adjusted accordingly?
• Possibility to contact weaning facility is given and contacts are documented	• Yes/No<br>• Documentation of contact frequency and results	

Attachment to: Kastrup M, Tittmann B, Sawatzki T, Gersch M, Vogt C, Rosenthal M, Rosseau S, Spies C. Transition from in-hospital ventilation to home ventilation: process description and quality indicators. GMS Ger Med Sci. 2017;15:Doc18. DOI: 10.3205/000259

10. Quality Indicator X

Patient Safety, Quality of Life and PRO

Dimension: Patient-Reported Outcomes

10.1 Cross-sectoral and -professional implementation of CIRS system, embedded in QRM
10.2 CIRS reports are regularly processed.
10.3 Physician orders in the patient record are up to date.
10.4 Assessment and documentation of quality of life
  10.4.1 At discharge
  10.4.2 1 month post-discharge
  10.4.3 Every 6 months post-discharge
  10.4.4 When quality of life decreases, a team meeting is ordered.
10.5 Number of patients receiving SAPV or AAPV (per the total number of treated patients in a calendar year)
10.6 Unplanned hospital admissions (amount of admissions and admission diagnosis) documented.
  10.6.1 Amount per patient
  10.6.2 Admission diagnosis
10.7 Number of deceased patients
  10.7.1 Died expectedly, being cared for in the outpatient setting
  10.7.2 Died unexpectedly in the outpatient setting
  10.7.3 Died in-hospital after transfer from the outpatient setting
10.8 Number of patients weaned successfully from mechanical ventilation (with or without NIV) and discharged into home environment
10.9 Number of patients discharged into
  10.9.1 Home environment
  10.9.2 Assisted living
  10.9.3 Residential community
  10.9.4 Nursing home
  10.9.5 Rehabilitation center

Reasoning and Definition:

- Ventilated patients, especially those with 100% dependency on the respirator, are a vulnerable population with high security requirements for their health care management.
- An outpatient warning system is hindered by deficiencies of cross-sectoral cooperation and lack of implementation.
- A CIRS (Critical Incident Reporting System) can aid in risk and quality management in order to avoid future mistakes.
- The quality of life of patients receiving outpatient mechanical ventilation has been scientifically evaluated and described as limited, depending on the underlying disorder (see [34]); if conclusions should be drawn to influence patient management, the quality of life must be regularly reassessed, considering the increasing number of patients receiving outpatient mechanical ventilation and/or of those with complex underlying disorders.
- There are disease specific and non-specific questionnaires to measure quality of life
  → Patient safety, individual quality of life and patient autonomy support must be the central focus of all persons involved in patient care.

Key Figures:

- CIRS is being implemented across all sectors and professions.
- Orders (especially medications, use of assistive devices, incl. accessories for ventilation, speech therapy, physical/occupational therapy, palliative care) are documented in the patient record/ePA and accessible to all personnel involved in care.
- Individual quality of life is assessed (e.g. vie the SF-36, SRI) before initiation of therapy and regularly thereafter – at least once a year – and documented in the ePA (standardized assessment).
- In case of decreasing quality of life, a case conference/ethics consult/team meeting is arranged.
- The multimodal therapeutic concept, planning of nursing care and supply of assistive devices will take patient autonomy resources into consideration; appropriate references are available in the ePA.

Key Figure	Degrees of Freedom	Comments
• CIRS is implemented and embedded in quality and risk management	• Yes/No	Reporting sector? What is being reported? Who reviews? Who has access? Which system? How many entries? Where does data flow lead? Who reacts?
• Reports are processed on a regular basis (monthly)	• Yes/No	
• Physician orders in the patient record are up to date	• Yes/No	Documentation in ePA
• Quality of life assessment and documentation (SF 36 – non-specific; SRI – for home mechanical ventilation):<br>• At discharge<br>• 1 month post-discharge<br>• Every 6 months post-discharge<br>• Team meeting is initiated when a decrease in QoL is found	• Results<br>• Team meeting has taken place, decisions are implemented	Documentation of assessment in ePA
• Number of patients receiving SAPV or AAPV (per total number of patients in calendar year)	• Patient w/ SAPV or AAPV/total no. of patients treated	
• Unplanned hospital admissions	• Amount per patient<br>• Admission diagnosis	Documentation in ePA
• Number of deceased patients	• Expected and deceased in outpatient setting<br>• Unexpected and deceased in outpatient setting<br>• Deceased in-hospital after transfer from outpatient setting	Documentation in ePA
• Number of patients who were successfully weaned (with or without NIV) and discharged into home environment	• Number of patients successfully weaned	Documentation in ePA
• Number of patients discharged into…	• Home environment<br>• Assisted living<br>• Residential community<br>• Nursing home<br>• Rehabilitation center	Documentation in ePA
